# Reference intervals for selected haematological and biochemical parameters among apparently healthy adults in different eco-geographical zones in Ghana

**DOI:** 10.1371/journal.pone.0245585

**Published:** 2021-01-20

**Authors:** Gabriel Abbam, Samuel Tandoh, Mary Tetteh, David Amoah Afrifah, Max Efui Annani-Akollor, Eddie-Williams Owiredu, Charles Gyasi, Constance Adu-Gyamfi, Benedict Sackey, Alexander Yaw Debrah, Otchere Addai-Mensah

**Affiliations:** 1 Department of Medical Diagnostics, Faculty of Allied Health Sciences, Kwame Nkrumah University of Science and Technology, Kumasi, Ghana; 2 University Clinic Laboratory, University of Education, Winneba, Ghana; 3 Department of Molecular Medicine, School of Medicine and Dentistry, Kwame Nkrumah University of Science and Technology, Kumasi, Ghana; Karolinska Institutet, SWEDEN

## Abstract

**Background:**

Due to the influence of gender, race/genetics, age, lifestyle habits and geography on the references intervals (RIs), the Clinical and Laboratory Standards Institute (CLSI) recommends the determination of population-specific RIs. Ghana continues to depend on pre-established RIs from other countries which poses the risk of misdiagnoses and wrong treatment. This study presents the haemato-biochemical RIs from four eco-geographical zones in Ghana.

**Methods:**

In this population-based cross-sectional study, a total of 1227 randomly selected healthy voluntary blood donors from the four eco-geographic zones (Coastal Savannah, Rain Forest, Savannah and Transitional) were enrolled and screened. Based on the CLSI Guidance Document C28A2992, the data of eligible participants were used to non-parametrically determine the RIs for the haemato-biochemical parameters at the 2.5^th^ and 97.5^th^ percentiles. Comparison of analytes by gender was done by Wilcoxon rank sum test and eco-geographic differences were assessed using the Kruskal-Wallis with the Dunn post hoc multiple comparison tests.

**Results:**

There were statistically significant differences in most of the haematological parameters (RBC, Hb, HCT, MCV, PLT, WBC; p-values <0.0001 and MCH; p-value = 0.007), and biochemical analytes (Urea, Cr, Trig, HDL-C, AST, ALT, ALP, GGT, BID, BIT, Prot-T and Albumin; p-values <0.0001) based on gender. Significant inter eco-geographic (intra-population) variations and substantial differences between the established RI and the RIs accompanying the analyzers used were also observed.

**Conclusion:**

This study reports significant inter-sex and inter-geographical differences in haemato-biochemical RIs in Ghana as well as differences in RIs with both the RIs accompanying the analyzers and those of other countries. Determining RIs representative of populations and including them in the report systems of laboratories to ensure effective and efficient healthcare service delivery is thus recommended.

## Introduction

Clinical laboratory tests are performed not only for health screening, diagnosis and management of disease, but also to monitor the progress of treatment [1]. The importance of these tests underscores the need for accurate and reliable results. Reference intervals (RIs) are threshold values within which a specified proportion of measurements from a healthy population would fall [2, 3]. They provide the basis of interpretation of laboratory results [4–6] and are thus an essential component of reporting laboratory test results [7].

Ghana’s health care delivery system has been evolving in recent years. Most requested laboratory tests in all clinics and hospitals in the country however still rely heavily on the manufacturers’ RIs (RIs accompanying the analyzers) which were largely derived from Caucasian populations [8–11]. Evidence suggests that RIs are influenced by gender, race/genetics, age and geographic origin of the population [8, 9, 12]. Apart from inter-population differences, we have demonstrated intra-population variability of RIs in Ghana [13]. Indeed, the reliance on RIs developed based on another population poses the risk of misdiagnoses which consequently results in wrong treatment. For this reason, the Clinical and Laboratory Standards Institute (CLSI) recommends the determination of population-specific RIs [6]. It is therefore critical for Ghana, a country with a diverse population demography and eco-geographic environment, to have population- and zone-specific laboratory RIs.

Against this background, this study, as part of an ongoing nationwide research aimed at establishing country- and region-specific haematological and biochemical reference intervals, reports the determination of haemato-biochemical RIs from four eco-geographical zones in Ghana.

## Materials and methods

### Ethical approval

The research protocol was approved by the Committee on Human Research, Publication and Ethics-KNUST (Reference number: CHRPE/AP/310/19), the 37 Military Hospital Institutional Review Board (Reference Number: 37MH-IRB IPN/300/2019) and Ghana Health Service Ethics Review Committee (GHS-ERC Number: GHS-ERC093/04/19). The objectives and benefits of the study were explained to all participants and written informed consent were obtained prior to enrollment in the study.

### Study design and site

A population-based cross-sectional study among apparently healthy blood donors was conducted from July 2019 to March 2020. The study included voluntary blood donors from different eco-geographic zones in Ghana. Ghana is a tropical country with warm and humid climates covering a land area of about 238,717 km^2^ and eco-geographically partitioned into four zones, namely Coastal Savannah, Rain Forest, Transitional and Savannah zones. The Coastal Savannah zone is about 12,732 km^2^ and covers predominantly Greater Accra and parts of Central and Volta regions, with average elevation between 20m and 150m above sea level. It has an annual mean temperature of 26°C-30°C, and mean rainfall of 750mm-850mm. The Rain Forest zone is about 61,651 km^2^ and covers the Western, parts of Central, Eastern, Ashanti and Brong Ahafo regions, with average elevation between 15m and 240m above sea level. It has thick vegetation which contributes to a high amount of rainfall (≥2000mm annually), rich soil types suitable for a wide range of crops cultivation and undulating lowlands with inselbergs. The Transitional zone has an area of about 65,152 km^2^ and covers parts of Eastern, Ashanti, Volta and Brong Ahafo regions and has an average elevation between 150m and 640m above sea level. It has an annual mean rainfall of about 1250mm-1750mm and mean temperature of 26°C-30°C. The climatic features and vegetation makes this zone more suitable for agriculture [14, 15]. The Savannah zone covers an area of about 99,182 km^2^, comprising Northern, Upper East and Upper West regions. It experiences one rainfall season annually (1,000mm-1,125mm) and the highest annual mean temperature of about 27°C-36°C. This zone has an average elevation between 180m and 300m above sea level and is the most sparingly populated and most of the populace practice subsistence farming [16]. Blood bank facilities within the four zones (37 Military Hospital-Accra (Coastal Savannah), Effia Nkwanta Regional Hospital-Sekondi/Takoradi (Rain Forest), Kwahu Government Hospital-Kwahu (Transitional) and Tamale Central Hospital-Tamale (Savannah)) were randomly selected as sampling sites.

### Reference population, enrolment and sample size

This study involved randomly selected healthy adult voluntary blood donors between 18 to 59 years old. Eligible participants were interviewed via a general health status questionnaire (adapted from the CLSI Guidance Document C28A2) [17]. Pregnant and breastfeeding mothers, obesity (BMI >29 kg/m^2^), evidence of medication use, use or abuse of alcohol and tobacco, presence of acute/chronic disease conditions, history of blood donation or transfusion within the last 3 months, surgery or hospitalization within the last 1 to 6 months, incomplete laboratory analysis results and any other confounding factors that may compromise the assessment of the analytes of interest were excluded from the study.

**Fig 1:**
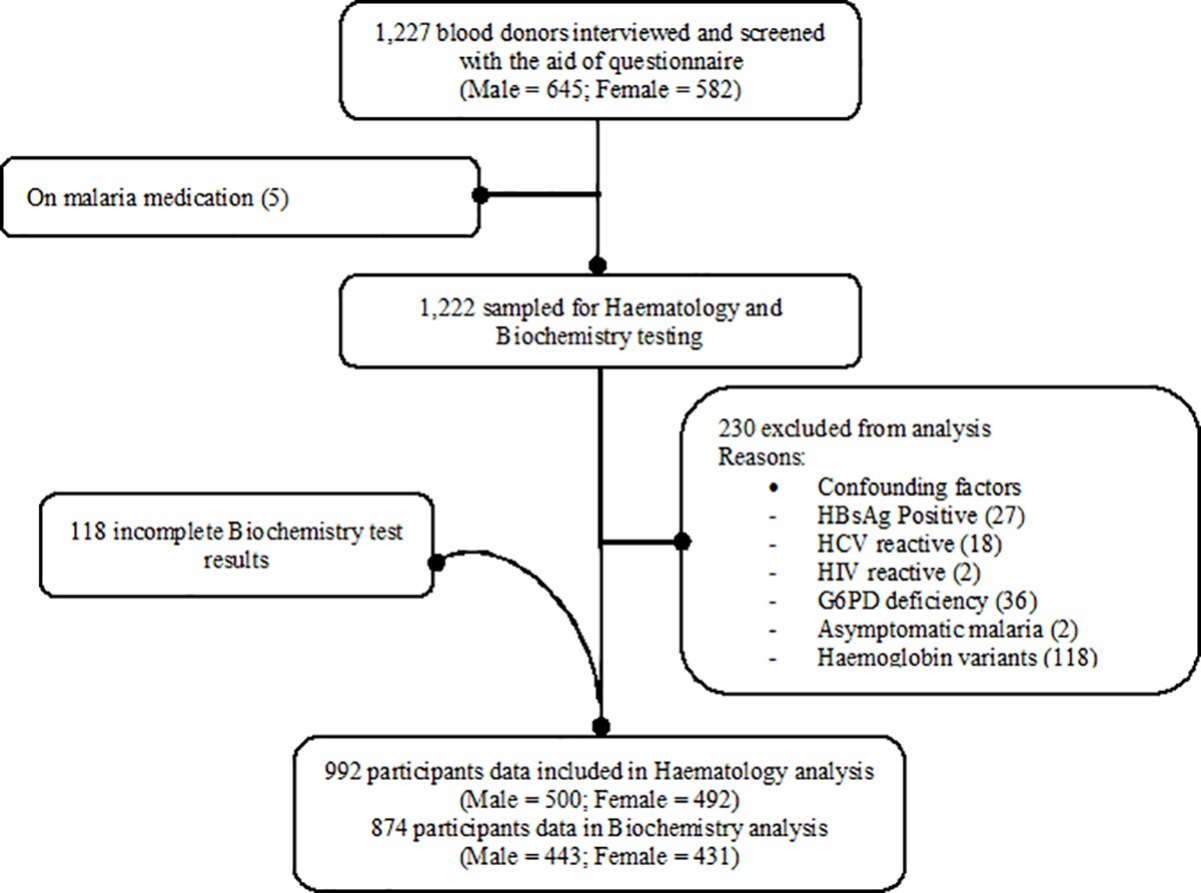
Flow chart of participant selection protocol.

According to the CLSI Guidance Document C28A2, a minimum of 120 participants are required in each group for non-parametric determination of RIs [17]. However, in an effort to improve the statistical power of the study, a total of 1227 healthy adult blood donors (at least 300 from each of the four eco-geographic zones) were invited to participate. Of the 1227 participants who were interviewed and screened, 235 were excluded due to possible confounding factors as shown in **Fig 1**. A total of 992 healthy blood donors were thus included in the final analyses. Biochemistry results were available for 874 participants. For the establishment of RIs based on gender to account for the differences in body physiology based on sex, males:500/433 and females: 492/431 were recruited for haematological/ biochemical RIs, respectively. For RIs based on co-geographical zones to account for the differences in geographical location, Coastal Savannah: 333/245, Rain Forest: 323/262, Savannah: 279/127, Transitional: 292/240 were recruited.

### Sample collection and laboratory assays

About 8ml of venous blood sample was collected from each participant in the morning between 8:00 AM and 11:00 AM. About 5ml of the sample was dispensed into EDTA tubes (Jactermac, Germany) and the remaining 3ml was dispensed into vacuum gel tubes (Jactermac, Germany) for haematology and biochemistry assays, respectively. The needle of the syringe was removed prior to dispensing the blood into tubes to avoid hemolysis. The samples were transported in a cold box to the laboratory within 3 hours for laboratory analyses. The samples in the gel tubes were spun at 4000rpm for 10 minutes to obtain the serum. Screening for confounding factors were performed and cases such as G6PD deficiency, asymptomatic or sub-clinical malaria, Hepatitis B and C, HIV, sickle cell and other abnormal haemoglobin variants were excluded (**Fig 1**).

Haematological analyses (red blood cell count (RBC), haemoglobin level (Hb), haematocrit (HCT), mean cell volume (MCV), mean cell haemoglobin (MCH), mean cell haemoglobin concentration (MCHC), red cell distribution widths (RDW-CV and RDW-SD), platelet count (PLT), white blood cell count (WBC), lymphocyte counts-absolute and percentage (LYM # and LYM %, respectively), monocyte count- absolute and percentage (MON # and MON %, respectively), neutrophil counts-absolute and percentage (NEU # and NEU %, respectively), eosinophil counts-absolute and percentage (EOS # and EOS %, respectively), and basophil counts-absolute and percentage (BAS # and BAS %, respectively) were performed within 8 hours of blood draw using YUMIZEN H500 (5-part differential) haematology auto analyzer (HORIBA ABX, France) and biochemical analyses (aspartate transaminase (AST), alanine transaminase (ALT), alkaline phosphatase (ALP), Gamma glutamyl transferase (GGT), direct bilirubin (BID), total bilirubin (BIT), albumin, total protein (Prot-T), total cholesterol (TChol), triglyceride (Trig), high density lipoprotein cholesterol (HDL-C), low density lipoprotein cholesterol (LDL-C), urea and creatinine levels) were performed using the DIALAB Autolyser according to the manufacturer’s instructions (DIALAB GmbH, Austria; https://www.dialab.at/en/products/instruments/clinical-analysers/autolyser/). The same type of analyzer was used in each study site. Daily calibration and maintenance of the analyzers were performed. Internal quality control (using low, normal and high QCs for hematological; normal and pathological QCs for biochemistry analyzers, respectively) was performed and analyses commenced only when all quality controls were within range. Aside from internal quality control, the lab participates in external quality assessment scheme by the United Kingdom International External Quality Assessment Scheme-UK IEQAS.

### Confounding factors assessments

Sickle cell screening for each participant was done using 2% sodium metabisulphite and haemoglobin phenotype was investigated using alkaline electrophoresis at pH of 8.6. The methaemoglobin reductase technique was used for G6PD screening. Screening for syphilis (InTec-One-step immunoassay; Xiamen Inc., China), hepatitis B and C (Biotech Co. Ltd; Guangzhou Wondfo; China) and HIV (first response HIV1&2 card test; Premier Medical Corporation Ltd-India and oral quick by Orasure Technologies, Inc, Bethlehem, PA, USA) were done by rapid diagnostic test kits. Malaria was investigated using both rapid diagnostic test kits (CareStartTM -ACCESSBIO, USA) and 10% Giemsa-stained thick film for microscopy

### Data management and statistical analysis

The study questionnaire was programmed into KoBoToolbox (https://www.kobotoolbox.org/), a web-based data collection software for ease of data collection and protection. Data was exported from the KoBoToolbox into Microsoft Excel Spreadsheet and verified. Stata version 14.1 (Stata Corp, College Park, TX, USA) was used for statistical analysis. The data was grouped based on gender and eco-geographical zones. The RIs were non-parametrically determined at 2.5th and 97.5th percentiles in accordance with the CLSI Guidance Document C28A2. Outliers estimated using the absolute difference between the most extreme distribution and the next value (D) and the Range (maximum-minimum) (R). Outliers were retained when D/R<0.33 [8]. Comparison of haemato-biochemical parameters by gender was performed by Wilcoxon rank sum test. Kruskal-Wallis tests with the Dunn post hoc tests were performed for differences between the four eco-geographical zones. A p-value of <0.05 was considered statistically significant.

## Results

### Sociodemographic characteristics of the study population

The 992 participants comprised 245 (24.7%) from the Coastal Savannah zone, 262 (26.41%) from the Rain Forest zone, 245 (24.7%) from the Savannah zone and 240 (24.19%) from the Transitional zone. The mean age of the entire study population was 27.0 ± 6.7 years old. Other sociodemographic characteristics of study population stratified by eco-geographic zones are shown in **Table 1**.

**Table 1:** Sociodemographic characteristics of study population by eco-geographic zones.

Variable	Total (N = 992)	Coastal Savannah (20m-150m)†	Rain Forest (15m-240m)†	Savannah (180m-300m)†	Transitional (150m-640m)†
Total; n (%)		n = 245 (24.7%)	n = 262 (26.41%)	n = 245 (24.7%)	n = 240 (24.19%)
**Mean Age (years)**	27.0 ± 6.7	29.8 ± 8.2	26.4 ± 7.0	26.9 ± 4.4	25.0 ± 5.7
Gender					
Male	500 (50.4%)	121 (49.4%)	135 (51.5%)	124 (50.6%)	120 (50%)
Female	492 (49.6%)	124 (50.6%)	127 (48.5%)	121 (49.4%)	120 (50%)
**Religion**					
Christian	800 (80.6%)	218 (89.0%)	248 (94.6%)	154 (62.9%)	180 (75%)
Islam	182 (18.3%)	25 (10.2%)	13 (5.0%)	89 (36.3%)	55 (22.9%)
Traditional	10 (1%)	2 (0.8%)	1 (0.4%)	2 (0.8%)	5 (2.1%)
**Educational Status**					
Basic	82(8.3%)	16 (6.5%)	16 (6.1%)	22 (9.0%)	28 (11.7%)
Secondary	261 (26.3%)	68 (27.8%)	79 (30.2%)	47 (19.2%)	67 (27.9%)
Tertiary	604 (60.9%)	147 (60.0%)	156 (59.5%)	166 (67.7%)	135 (56.2%)
Postgraduate	45 (4.5%)	14 (5.7%)	11 (4.2%)	10 (4.1%)	10 (4.2%)
**Marital Status**					
Single	673 (67.8%)	174 (71.0%)	197 (75.2%)	130 (53.1%)	172 (71.7%)
Married	304 (30.6%)	64 (26.1%)	64 (24.4%)	111 (45.3%)	65 (27.1%)
Divorced	6 (0.6%)	3 (1.2%)	1 (0.4%)	1 (0.4%)	1 (0.4%)
Widowed	9 (0.9%)	4 (1.6%)	0 (0%)	3 (1.2%)	2 (0.8%)
**Occupation Status**					
Unemployed	444 (44.8%)	89 (36.3%)	127 (48.5%)	102 (41.6%)	126 (52.5%)
Employed	548 (55.2%)	156 (63.7%)	135 (51.5%)	143 (58.4%)	114 (47.5%)
*Formal*	*292 (29*.*4%)*	*89 (57*.*1%)*	*58 (43*.*0%)*	*60 (42*.*0%)*	*85 (74*.*6%)*
*Informal*	*256 (25*.*8%)*	*67 (42*.*9%)*	*77 (57*.*0%)*	*83 (58*.*0%)*	*29 (25*.*4%)*

† Altitude of the various zones

### Haematology reference intervals by gender

RBC count, Hb level, HCT, MCV, MCH, percentage and absolute eosinophil counts were significantly higher in males than females (p<0.05). However, RDW-CV, platelets and WBC were significantly higher in females than in males (p<0.0001) (**Table 2 and S1 Table**).

**Table 2:** Haematology reference intervals by gender.

Parameters	Unit	Combined RIs			Males			Females			p-value
		N	Median	Reference values	N	Median	Reference values	N	Median	Reference values	
RBC	10^6^/μL	982	5.03	3.97–6.28	494	5.31	4.20–6.47	488	4.69	3.83–5.71	<0.0001
Hb	g/dL	985	14.1	10.60–17.34	500	15.2	12.35–17.75	485	13.2	10.22–15.50	<0.0001
HCT	%	990	40.8	31.00–50.70	500	44.8	32.50–51.50	490	37.9	29.00–45.30	<0.0001
MCV	μm^3^	956	81.8	68.20–95.00	472	82.5	67.30–96.50	484	80.8	68.40–92.00	<0.0001
MCH	pg	958	28.4	23.20–32.50	486	28.6	23.30–32.70	472	28.3	23.10–32.20	0.0077
MCHC	g/dL	863	34.3	31.70–36.70	421	34.4	31.40–36.60	442	34.3	31.80–36.9	0.655
RDW-CV	%	986	11.8	8.70–14.40	499	11.4	8.55–13.80	487	12	8.70–14.94	<0.0001
RDW-SD	μm^3^	943	41.2	30.20–52.90	458	41.25	29.78–53.58	485	41.2	31.10–52.40	0.1813
PLT	10^3^/μL	967	255	140.20–384.00	493	237	127.10–357.30	474	269	158.88–405.00	<0.0001
WBC	10^3^/μL	968	5.2	3.16–7.73	493	4.98	3.08–7.67	475	5.39	3.28–7.85	<0.0001
LYM #	10^3^/μL	967	2.2	1.31–3.38	484	2.22	1.19–3.34	483	2.2	1.34–3.41	0.9316
LYM %	%	969	45.9	30.55–62.40	489	45.9	30.13–63.3	480	45.7	30.7–61.20	0.5084
MON #	10^3^/μL	954	0.4	0.20–0.68	485	0.4	0.19–0.70	469	0.39	0.20–0.66	0.7394
MON %	%	947	8	4.70–12.33	477	8	4.50–12.30	470	7.9	5.06–12.42	0.6892
NEU #	10^3^/μL	973	1.97	0.94–3.52	491	1.94	0.85–3.56	482	2.01	0.99–3.52	0.1228
NEU %	%	957	41.6	24.30–56.40	483	41.1	23.90–56.40	474	41.75	25.59–56.31	0.054
EOS #	10^3^/μL	821	0.08	0.02–0.20	404	0.09	0.02–0.20	417	0.07	0.02–0.19	0.002
EOS %	%	788	1.6	0.40–3.60	385	1.6	0.40–3.60	403	1.5	0.364–3.59	0.0019
BAS #	10^3^/μL	952	0.07	0.03–0.13	479	0.07	0.03–0.13	473	0.07	0.03–0.12	0.6964
BAS %	%	961	1.5	0.70–2.60	484	1.5	0.70–2.50	477	1.5	0.70–2.60	0.202

RBC: Red Blood Cells; Hb: Haemoglobin; HCT: Haematocrit; MCV: Mean Cell Volume, MCH: Mean Cell Haemoglobin; MCHC: Mean Cell Haemoglobin Concentration; RDW-CV: Red cell Distribution Width-Coefficient of Variation; RDW-SD: Red cell Distribution Width-Standard Deviation; PLT: Platelet count; WBC: White Blood Cells; LYM: Lymphocyte; MON: Monocyte; NEU: Neutrophil; EOS: Eosinophil; BAS: Basophil; ‘

#: Absolute

### Haematology reference intervals by eco-geographical zones

The Coastal Savannah zone presented with lower RBC count, Hb level, HCT and RDW-CV but higher absolute neutrophils among both males and females whereas the Transitional zone recorded the highest RBC count and MCHC. The Savannah had a higher RBC count relative to the Coastal Savannah and Rain Forest zones and a higher MON#, BAS# and BAS% compared to the Rain Forest zone. Hb level was higher in the Savannah zone compared to the Coastal Savannah zone. The Rain Forest recorded a higher RBC count and Hb level compared to the Coastal Savannah zone, and a higher MCV compared to the Savannah and Transitional zones. (**Table 3 and S2 Table**).

**Table 3:** Haematology reference intervals by eco-geographical zones.

Sex	Males				Females			
Parameters	N	Median	Ref. Values	90% CI	N	Median	Ref. Values	90% CI
**Coastal Savannah Zone**								
RBC	121	5.05	4.08–6.17	3.94–4.3; 5.68–6.39	123	4.51	3.66–5.37	3.94–4.3; 5.68–6.39
Hb	121	14.6	11.82–16.50	10.60–12.43; 16.1–17	123	12.5	10.01–14.69	10.60–12.43; 16.1–17
HCT	121	42.6	31.00–52.10	27.6–32.1; 49.9–54.1	124	36.5	26.40–43.70	27.6–32.1; 49.9–54.1
MCV	97	83.7	65.70–98.00	64.4–66.7; 96.9–98.7	119	80.6	65.70–96.20	64.4–66.7; 96.9–98.7
MCH	118	28.9	23.40–32.40	22.7–23.8; 32.0–34.2	116	28.2	23.00–32.80	22.7–23.8; 32.0–34.2
MCHC	59	33	31.10–37.20	31.1–31.2; 36.5–37.2	104	34.5	31.30–37.30	31.1–31.2; 36.5–37.2
RDW-CV	121	10	8.70–12.99	8.3–8.8; 12.4–14	123	11.5	8.51–14.58	8.3–8.8; 12.4–14
RDW-SD	82	44.75	26.50–55.40	26.5–28.5; 55.4–55.4	117	38.6	27.00–55.40	26.5–28.5; 55.4–55.4
PLT	121	240	135.55–353.95	108–152.19; 335.26–406	117	274	161.85–421.30	108–152.19; 335.26–406
WBC	119	5.04	3.42–8.09	2.36–3.68; 7.13–8.18	121	5.38	3.44–8.01	3.24–3.62; 7.57–8.05
LYM #	117	2.19	1.37–3.15	1.09–1.46; 2.99–3.34	120	2.32	1.44–3.49	1.24–1.60; 3.38–3.59
LYM %	119	44.2	28.50–62.90	28.1–31.23; 56.17–64.1	124	45.7	28.30–58.11	25.7–31.69; 56.15–63.4
MON #	115	0.42	0.21–0.74	0.13–0.25; 0.68–0.74	114	0.43	0.18–0.67	0.12–0.26; 0.61–0.72
MON %	114	8.4	4.80–12.54	4.7–5.70; 12.00–13.1	117	8.1	5.10–12.82	4.9–5.63; 11.7–13.2
NEU #	118	2.05	1.06–3.75	0.99–1.34; 3.31–3.94	121	2.15	1.09–3.52	1.02–1.33; 3.40–3.91
NEU %	118	42.55	25.37–59.10	22.4–27.73; 56.33–59.3	123	41.3	28.95–57.49	25–31.66; 56.15–60.6
EOS #	91	0.08	0.01–0.21	0.01–0.03; 0.18–0.21	103	0.07	0.02–0.20	0.01–0.03; 0.17–0.22
EOS %	87	1.7	0.20–3.28	0.1–0.59; 3.11–3.7	103	1.4	0.30–3.64	0.2–0.47; 3.3–3.7
BAS #	118	0.08	0.03–0.13	0.03–0.04; 0.13–0.13	118	0.08	0.03–0.13	0.03–0.05; 0.12–0.13
BAS %	120	1.55	0.60–2.50	0.5–0.8; 2.37–2.7	121	1.4	0.70–2.50	0.5–0.8; 2.29–2.7
**Rain Forest**								
RBC	135	5.24	4.09–6.25	4.03–4.36; 6.09–6.64	125	4.67	3.83–5.73	3.7–4.01; 5.55–6.49
Hb	135	15.2	10.76–17.36	9.4–12.5; 17.11–18.6	123	13.1	9.57–15.38	9.4–10.84; 15.2–16.8
HCT	135	44.4	33.20–51.90	29.6–36.3; 49.7–54.9	125	38.1	29.00–45.00	26.7–31.5; 43.4–50.6
MCV	134	84.4	71.10–95.80	66.0–73.4; 91.6–98.8	125	82.3	69.30–90.80	65.4–72.2; 88.8–93.5
MCH	132	28.9	23.10–32.90	22.5–24.7; 31.8–34.3	122	28.2	22.60–32.10	22.6–24.2; 31.2–33.0
MCHC	129	34.3	31.60–36.60	31.1–32.1; 36.2–37.3	121	34.3	31.70–36.50	31.4–32.3; 36.1–36.7
RDW-CV	135	11.3	8.44–13.80	8.1–8.69; 13.4–14.4	125	12	8.7–14.49	8.4–8.95; 13.8–16.4
RDW-SD	134	39.9	30.84–51.40	28.6–32.66; 48.56–51.4	127	41.2	31.10–49.56	31.1–31.9; 47.16–54.4
PLT	134	231.5	132.50–346.25	111–153.73; 337.32–358	126	260.5	152.53–398.85	150–171.87; 369.13–411
WBC	134	4.86	3.03–7.34	2.87–3.21; 6.71–8.16	122	5.45	3.17–7.43	2.89–3.59; 7.12–8.27
LYM #	134	2.19	1.09–3.56	0.91–1.25; 3.09–3.72	124	1.95	1.06–3.23	0.91–1.34; 3.09–3.64
LYM %	131	46.4	33.40–63.30	26.8–36.85; 63.3–63.3	121	45.2	31.78–61.30	27.6–34.5; 58.3–62.4
MON #	134	0.33	0.19–0.66	0.16–0.2; 0.61–0.69	127	0.36	0.21–0.67	0.18–0.21; 0.63–0.68
MON %	131	7.9	3.63–11.27	3.6–4.47; 10.7–12.4	121	7.8	4.40–12.27	3.9–5.2; 10.53–12.7
NEU #	134	1.83	0.78–3.11	0.4–0.97; 3.01–3.75	124	1.98	0.99–3.70	0.76–1.06; 3.03–3.75
NEU %	128	40.25	23.75–56.17	21.6–24.1; 52.48–56.3	119	43.7	25.20–54.30	21.6–29.1; 53.87–55.7
EOS #	119	0.08	0.01–0.19	0.01–0.02; 0.17–0.2	111	0.07	0.02–0.18	0.01–0.02; 0.15–0.19
EOS %	116	1.65	0.29–3.70	0.2–0.51; 3.4–3.8	110	1.45	0.38–3.62	0.1–0.5; 3.13–3.8
BAS #	127	0.07	0.02–0.13	0.02–0.03; 0.12–0.13	123	0.07	0.02–0.11	0.02–0.02; 0.1–0.12
BAS %	125	1.5	0.60–2.60	0.3–0.85; 2.4–2.7	121	1.4	0.50–2.69	0.5–0.6; 2.4–2.7
**Savannah Zone**								
RBC	121	5.52	4.29–6.66	4.12–4.75; 6.51–6.74	121	4.81	3.66–5.73	3.57–3.99; 5.39–6.02
Hb	124	15.75	12.73–18.16	12.1–13; 17.4–18.8	121	13.4	10.31–15.50	9.4–10.94; 15.2–16.8
HCT	124	45.2	36.20–52.40	35.8–38.6; 50.6–54.7	121	38.7	29.90–46.30	27.0–31.4; 43.8–49.0
MCV	123	81.6	68.20–94.30	65.4–71.1; 89.7–98.3	121	80.7	70.40–91.60	69.3–72.2; 88.7–95.7
MCH	121	28	23.10–33.20	22.6–24.4; 32.0–33.6	121	28.1	23.10–32.10	22.8–24.3; 31.3–32.7
MCHC	122	34.5	32.30–36.60	31.8–32.8; 35.8–36.8	113	34.2	32.00–35.80	31.2–32.7; 35.7–36.5
RDW-CV	123	12	8.71–14.30	8.4–9.04; 13.86–15.5	121	12.2	8.80–15.40	8.4–8.9; 14.88–16.2
RDW-SD	124	40.3	30.20–49.45	29.4–31.9; 47.45–52.9	121	41.2	31.10–47.90	30.2–32.8; 47–47.9
PLT	120	237	125.18–375.95	100–140.95; 344.36–410	117	260	158.00–375.70	128–165.33; 361.87–423
WBC	123	4.98	2.81–7.71	2.38–3.22; 7.29–7.83	118	5.22	3.04–7.67	2.8–3.52; 7.21–7.85
LYM #	118	2.17	1.37–3.34	0.98–1.48; 3.03–3.38	119	2.17	1.46–3.31	1.43–1.51; 3.1–3.38
LYM %	122	45.9	29.23–61.00	27.4–35.2; 58.5–64.1	115	44.5	29.03–64.20	28.4–30.97; 62.79–65.9
MON #	119	0.41	0.17–0.72	0.17–0.21; 0.66–0.74	113	0.38	0.18–0.66	0.17–0.24; 0.59–0.66
MON %	116	8	4.40–12.41	3.1–6; 11.79–12.8	116	7.8	4.87–11.88	3.1–5.62; 10.8–12.9
NEU #	121	1.89	0.71–3.33	0.49–0.88; 3.19–3.95	118	2	0.49–3.75	0.49–1.06; 3.35–3.95
NEU %	122	39.85	22.23–55.63	21.3–25.58; 54.1–58.2	113	42.5	24.30–59.45	23.7–26.4; 53.81–60.3
EOS #	95	0.09	0.02–0.20	0.01–0.03; 0.19-.21	99	0.08	0.02–0.20	0.01–0.03; 0.18–0.2
EOS %	85	1.7	0.50–3.60	0.4–0.7; 3.4–3.8	90	1.5	0.33–3.27	0.3–0.50; 2.6–3.6
BAS #	121	0.08	0.03–0.13	0.03–0.04; 0.12–0.13	115	0.08	0.04–0.12	0.03–0.05; 0.12–0.13
BAS %	123	1.6	0.80–2.50	0.5–0.9; 2.35–2.7	118	1.6	0.80–2.70	0.8–1; 2.6–2.7
**Transitional Zone**								
RBC	117	5.59	4.54–6.39	4.29–4.82; 6.29–6.53	119	4.84	4.01–5.78	3.98–4.08; 5.61–6.09
Hb	120	15.8	12.61–18.00	11–13.40; 17.7–18.5	118	13.5	11.40–15.91	10.7–11.92; 15.7–16.3
HCT	120	46	37.10–51.30	34.3–38.5; 51.0–53.0	120	39.1	30.60–46.20	27.3–32.9; 45.2–47.7
MCV	118	82	67.40–91.90	65.5–71.4; 91.0–94.2	119	80.1	68.30–90.70	65.9–70.7; 88.5–94.5
MCH	115	28.5	23.90–32.70	22.7–24.9; 32.1–34.1	113	28.6	23.80–32.20	22.9–24.5; 31.3–33.5
MCHC	111	34.7	32.10–36.60	31.8–33.0; 36.4–37.1	104	34.5	32.10–37.20	31.9–32.3; 36.5–37.5
RDW-CV	120	12.05	8.70–13.70	8.4–8.93; 13.6–14.8	118	12	8.80–15.01	8.7–8.92; 14.3–15.5
RDW-SD	118	42	30.20–49.43	30.2–32.8; 47.4–50.4	120	40.3	31.12–51.16	30.2–31.9; 47.4–52.4
PLT	118	234	99.98–359.36	99–142.96; 342.78–387	114	281.5	148.00–407.00	136–186.99; 370.01–426
WBC	117	5.18	3.18–7.69	2.45–3.70; 7.15–8.18	114	5.48	3.30–8.23	2.97–3.80; 7.50–8.34
LYM #	115	2.34	1.42–3.60	1.14–1.57; 3.32–3.67	120	2.27	1.30–3.61	1.12–1.44; 3.29–3.67
LYM %	117	47.6	30.20–61.26	25.7–33.33; 59.65–65.2	120	46.9	32.81–59.20	31.3–34.15; 57.37–61.1
MON #	117	0.41	0.20–0.71	0.17–0.22; 0.65–0.74	115	0.42	0.19–0.67	0.19–0.22; 0.64–0.74
MON %	116	7.75	4.69–12.33	3.2–5.02; 11.88–13	116	8.4	4.62–12.91	3.6–5.3; 12.27–13.1
NEU #	118	2.01	1.03–3.72	0.91–1.12; 3.35–3.91	119	1.93	0.91–3.39	0.67–1.03; 3.17–3.77
NEU %	115	40	23.76–59.34	21.5–25.62; 55.96–60.6	119	40.5	25.20–56.40	22.8–27.68; 53.06–60.6
EOS #	99	0.08	0.02–0.21	0.01–0.03; 0.2–0.22	104	0.07	0.02–0.19	0.01–0.02; 0.17–0.22
EOS %	97	1.6	0.34–3.70	0.2–0.6; 3.36–3.8	100	1.6	0.35–3.65	0.2–0.5; 3.04–3.8
BAS#	113	0.07	0.03–0.13	0.03–0.05; 0.12–0.13	117	0.07	0.04–0.11	0.03–0.04; 0.1–0.12
BAS %	116	1.5	0.60–2.51	0.5–0.9; 2.3–2.6	117	1.6	0.90–2.51	0.7–1; 2.2–2.7

RBC: Red Blood Cells; Hb: Haemoglobin; HCT: Haematocrit; MCV: Mean Cell Volume, MCH: Mean Cell Haemoglobin; MCHC: Mean Cell Haemoglobin Concentration; RDW-CV: Red cell Distribution Width-Coefficient of Variation; RDW-SD: Red cell Distribution Width-Standard Deviation; PLT: Platelet count; WBC: White Blood Cells; LYM: Lymphocyte; MON: Monocyte; NEU: Neutrophil; EOS: Eosinophil; BAS: Basophil

#: Absolute

### Biochemistry reference intervals by gender

Analytes of the liver function (AST, ALT, ALP, GGT, BID, BIT, Alb and Prot-T), serum urea, creatinine, Trig and LDL-C were significantly higher in males than in females (p<0.0001). Females however had a significantly higher HDL-C compared to males (p<0.0001) (**Table 4 and S3 Table**).

**Table 4:** Biochemistry reference intervals by gender.

Parameters	Unit	Combined			Males			Females			p-value
		N	Median	Reference values	N	Median	Reference values	N	Median	Reference values	
AST	U/L	874	22.04	10.49–33.77	443	23.64	11.72–34.09	431	20.88	9.57–31.38	<0.0001
ALT	U/L	852	20.73	6.60–34.50	424	21.79	8.02–35.30	428	19.93	5.19–31.89	<0.0001
ALP	U/L	874	88.10	52.47–117.46	443	102.96	63.51–118.68	431	73.79	50.99–99.24	<0.0001
GGT	U/L	867	30.27	12.22–51.71	437	33.63	14.48–53.66	430	28.26	11.61–43.79	<0.0001
BID	mg/dL	871	0.144	0.037–0.309	442	0.171	0.032–0.310	429	0.118	0.044–0.305	<0.0001
BIT	mg/dL	865	0.907	0.542–1.169	437	0.934	0.543–1.178	428	0.876	0.540–1.159	<0.0001
Albumin	g/dL	869	4.38	3.82–4.86	441	4.49	3.83–4.90	428	4.29	3.77–4.76	<0.0001
Prot-T	g/dL	851	7.39	6.30–8.23	431	7.50	6.26–8.25	420	7.31	6.31–8.19	<0.0001
TChol	mg/dL	869	166.40	128.0–207.9	439	169.20	126.0–207.6	430	162.80	128.7–208.0	0.0578
Trig	mg/dL	872	84.50	43.4–126.4	443	89.40	42.2–129.5	429	80.80	44.3–119.5	0.0017
HDL-C	mg/dL	871	54.30	39.00–72.03	443	51.93	38.57–69.10	428	56.35	39.59–73.20	<0.0001
LDL-C	mg/dL	871	96.6	58.9–133.0	442	99.80	58.2–134.4	429	93.40	59.4–130.6	0.0002
Urea	mg/dL	863	19.91	13.20–23.01	437	20.42	14.00–23.03	426	18.64	12.44–22.83	<0.0001
Creatinine	mg/dL	825	0.891	0.594–1.175	440	0.941	0.605–1.192	385	0.820	0.580–1.093	<0.0001

AST: Aspartate transaminase; ALT: Alanine transaminase; ALP: Alkaline Phosphatase; GGT: Gamma-Glutamyl Transferase; BID: Direct bilirubin; BIT: Indirect bilirubin; Prot-T; Protein; TChol: Total cholesterol; Trig: Triglyceride; HDL-C: high-density lipoprotein; LDL-C: low-density lipoprotein

### Biochemistry reference intervals by eco-geographical zones

Coastal Savannah zone had higher BID, TChol and Trig but lower ALT, BIT, Albumin and Prot-T compared to Rain Forest zone. Savannah zone recorded a higher ALT, Albumin, Prot-T and HDL-C but lower TChol, Trig, LDL-C and urea compared to Coastal Savannah zone. Transitional zone presented with higher ALT, BIT, Trig and creatinine compared to the Rain Forest zone (**Table 5 and S4 Table**).

**Table 5:** Biochemistry reference intervals by eco-geographical zones.

Sex	Males				Females			
Parameters	N	Median	Ref. Values	90% CI	N	Median	Ref. Values	90% CI
**Coastal Savannah Zone**								
AST	121	23.04	11.49–35.20	6.65–15.57; 32.59–37.42	124	20.93	8.48–34.03	7.65–10.17; 31.45–36.73
ALT	110	17.66	6.92–37.26	4.76–8.90; 34.84–40.49	123	15.78	3.87–35.77	2.63–5.04; 30.00–39.62
ALP	121	103.74	65.42–121.00	57.38–71.28; 118.31–123.40	124	75.25	52.29–97.90	44.52–53.30; 96.55–111.09
GGT	115	30.86	14.58–54.66	5.21–15.60; 54.04–55.00	123	25.61	12.19–47.69	10.02–13.48; 43.13–54.89
BID	121	0.184	0.019–0.314	0.006–0.036; 0.311–0.318	124	0.177	0.043–0.307	0.022–0.062; 0.300–0.365
BIT	119	0.895	0.452–1.158	0.408–0.528; 1.118–1.172	123	0.827	0.524–1.198	0.433–0.544; 1.098–1.201
Albumin	119	4.36	3.82–4.84	3.59–3.93; 4.78–4.95	121	4.32	3.64–4.79	3.57–3.89; 4.70–4.86
Prot-T	115	7.25	6.19–8.30	6.14–6.33; 8.21–8.42	115	7.09	6.21–8.26	6.19–6.32; 8.13–8.29
Tchol	120	175.1	115.5–208.4	111.0–129.4; 201.0–209.4	124	175.4	119.4–208.7	115.8–129.5; 207.8–209.3
Trig	121	93.9	36.1–137.3	30.4–45.7; 129.7–149.7	123	82	43.1–126.2	35.8–46.1; 118.8–140.5
HDL-C	121	52.52	38.85–71.34	36.04–39.85; 68.68–74.18	122	57.09	37.56–75.59	35.76–40.00; 71.69–77.48
LDL-C	121	103.5	50.2–138.8	46.7–62.6; 135.3–141.1	123	105.3	57.4–133.3	48.4–59.5; 130.3–134.3
Urea	119	20.89	14.35–23.73	13.38–15.30; 22.91–25.93	122	19.45	12.24–23.07	11.18–13.33; 22.61–23.07
Creatinine	120	0.877	0.617–1.146	0.606–0.655; 1.070–1.230	84	0.753	0.605–1.123	0.587–0.616; 1.005–1.249
**Rain Forest Zone**								
AST	135	22.13	12.67–35.54	10.26–14.72; 33.83–36.45	127	21.84	10.94–30.46	10.57–13.64; 27.70–35.73
ALT	130	19.72	10.30–38.88	7.37–11.30; 32.50–40.33	125	20.94	10.01–29.82	7.41–11.03; 29.29–30.70
ALP	135	104.25	60.21–118.98	55.46–72.47; 116.84–121.64	127	76.22	48.08–99.34	43.29–54.09; 95.75–106.73
GGT	135	29.67	14.07–54.33	8.88–17.83; 51.97–55.07	127	31.28	11.31–45.34	7.50–13.63; 41.09–47.18
BID	134	0.194	0.021–0.312	0.015–0.048; 0.302–0.326	125	0.095	0.045–0.296	0.026–0.055; 0.225–0.314
BIT	134	0.971	0.587–1.194	0.554–0.607; 1.177–1.200	126	0.931	0.554–1.146	0.473–0.602; 1.109–1.169
Albumin	135	4.54	3.66–4.88	3.59–3.83; 4.78–4.95	127	4.32	3.86–4.80	3.62–3.96; 4.72–4.95
Prot-T	129	7.5	6.24–8.29	6.14–6.47; 8.21–8.30	126	7.41	6.66–8.19	6.25–6.92; 8.03–8.23
Tchol	134	168.3	125.8–207.6	118.2–130.7; 204.4–210.7	126	165.5	127.0–203.1	120.4–138.1; 195.1–222.4
Trig	135	81.6	43.2–123.2	40.6–46.1; 120.3–130.5	126	76.4	45.6–116.0	41.7–52.3; 106.9–150.3
HDL-C	135	53.04	37.10–68.02	35.75–40.86; 64.65–75.46	126	54.11	40.05–72.16	35.76–44.55; 67.89–75.26
LDL-C	134	100.6	59.6–134.8	46.0–67.0; 131.6–137.5	126	95.5	59.6–127.5	57.1–68.2; 115.9–143.6
Urea	133	20.58	14.80–23.04	13.03–15.46; 22.92–23.79	126	18.54	12.44–22.78	11.48–13.52; 22.07–24.64
Creatinine	134	0.925	0.603–1.227	0.534–0.649; 1.177–1.276	125	0.816	0.593–1.070	0.417–0.621; 1.050–1.206
**Savannah Zone**								
AST	67	24.5	11.22–34.05	10.38–14.15; 30.43–34.36	60	19.39	11.86–33.06	11.15–13.64; 28.82–36.10
ALT	65	25.69	5.78–33.17	3.90–8.87; 31.84–33.41	60	19.33	4.83–30.29	2.90–11.41; 27.25–30.65
ALP	67	103.19	62.65–117.03	62.48–69.34; 112.81–117.29	60	66.46	51.07–96.54	59.47–53.13; 88.49–98.32
GGT	67	34.34	10.65–49.45	9.95–17.08; 42.51–51.62	60	26.73	8.91–35.19	8.51–15.11; 32.47–37.46
BID	67	0.134	0.057–0.301	0.044–0.071; 0.280–0.301	60	0.103	0.059–0.294	0.055–0.069; 0.246–0.316
BIT	65	0.882	0.481–1.141	0.390–0.583; 1.066–1.158	59	0.918	0.622–1.154	0.622–0.680; 1.097–1.178
Albumin	67	4.5	3.93–4.89	3.90–4.16; 4.76–4.94	60	4.35	3.91–4.75	3.77–4.07; 4.67–4.76
Prot-T	67	7.56	6.45–8.27	6.24–6.91; 8.06–8.27	59	7.34	6.20–8.22	6.17–6.49; 8.07–8.30
Tchol	66	158.9	126.3–208.9	122.3–132.4; 186.7–225.5	60	159.6	130.3–208.9	121.9–141.7; 192.6–209.1
Trig	67	68.8	39.8–114.1	38.9–42.7; 102.2–127.3	60	79.6	44.6–117.5	43.4–57.2; 105.2–119.0
HDL-C	67	55.97	42.38–71.12	39.57–45.27; 68.78–72.65	60	61.15	43.50–74.21	38.57–49.55; 72.66–75.46
LDL-C	67	92	56.6–125.9	54.2–66.4; 109.9–132.1	60	85.3	59.5–130.3	58.7–61.4; 114.0–134.23
Urea	66	19.81	12.86–23.27	11.76–14.06; 22.37–23.80	58	18.52	12.20–22.37	11.34–13.87; 21.72–22.42
Creatinine	66	0.914	0.491–1.045	0.504–0.553; 1.033–1.074	59	0.791	0.519–1.073	0.504–0.553; 1.033–1.074
**Transitional Zone**								
AST	120	24.99	9.12–32.61	7.89–14.86; 31.44–34.62	120	20.54	7.95–30.49	5.64–10.65; 28.84–31.88
ALT	119	25.26	9.56–34.11	6.24–12.49; 33.29–35.13	120	21.51	5.55–34.63	0.75–9.43; 30.48–37.14
ALP	120	98.44	63.52–118.37	51.70–70.40; 116.33–119.17	120	72.55	51.12–105.04	44.65–52.80; 98.57114.40
GGT	120	37.9	14.50–50.42	10.68–20.74; 49.53–51.58	120	28.22	11.68–41.03	10.09–14.67; 38.57–44.63
BID	120	0.149	0.042–0.281	0.031–0.058; 0.248–0.307	120	0.107	0.037–0.302	0.015–0.047; 0.278–0.311
BIT	119	0.973	0.620–1.182	0.594–0.686; 1.149–1.186	120	0.833	0.518–1.118	0.435–0.568; 1.067–1.179
Albumin	120	4.52	4.00–4.94	3.98–4.06; 4.88–4.97	120	4.21	3.75–4.83	3.56–3.87; 4.66–4.92
Prot-T	120	7.51	6.79–8.20	6.43–7.02; 8.14–8.22	120	7.26	6.86–8.18	6.72–6.93; 8.01–8.30
Tchol	119	169.4	144.7–195.4	141.2–149.8; 190.1–208.4	120	162.5	132.6–207.9	131.0–135.8; 201.7–209.2
Trig	120	98.3	52.7–120.5	50.3–61.9; 116.6–129.7	120	87.3	44.6–119.4	41.4–50.3; 116.5–128.4
HDL-C	120	49.94	37.28–67.23	35.69–39.59; 61.78–69.06	120	54.75	41.00–71.45	39.50–43.42; 69.42–76.43
LDL-C	120	100.4	73.3–130.8	46.4–75.0; 117.8–134.3	120	88.2	62.9–125.8	60.0–67.1; 122.5–138.9
Urea	119	20.14	13.44–23.01	12.53–14.25; 22.47–23.18	120	18.32	12.44–23.00	12.36–13.51; 22.21–26.19
Creatinine	120	1.005	0.850–1.211	0.422–0.897; 1.180–1.262	117	0.884	0.603–1.107	0.511–0.640; 1.073–1.128

AST: Aspartate transaminase; ALT: Alanine transaminase; ALP: Alkaline Phosphatase; GGT: Gamma-Glutamyl Transferase; BID: Direct bilirubin; BIT: Indirect bilirubin; Prot-T; Protein; TChol: Total cholesterol; Trig: Triglyceride; HDL-C: high-density lipoprotein; LDL-C: low-density lipoprotein

### Percentage out of range of the established haematological RIs compared to accompanying RIs

The percentage out of range (OOR) values of the study RIs were computed. **Tables 6** and **7** show the proportion of normal Ghanaian adults whose haematology and biochemistry laboratory results would have been described as abnormal when the accompanying RIs provided by HORIBA (haematological) and DIALAB (biochemistry) are used.

**Table 6:** Out of Range (OOR) haematological values based on comparison with HORIBA values.

Parameter	Unit	Male		Female	
		HORIBA values	%OOR	HORIBA values	%OOR
**RBC**	10^6^/μL	4.20–6.00	14.2	3.80–5.20	19.3
**Hb**	g/dL	13.0–17.0	15.2	11.5–15.2	16.1
**HCT**	%	39.0–52.0	17.2	35.0–46.0	24.6
**MCV**	μm^3^	76.0–100.0	20.8	77.0–97.0	29.1
**MCH**	pg	26.0–34.0	14.4	26.0–34.0	22.4
**MCHC**	g/dL	32. 0–35.0	42.8	32. 0–35.0	35.8
**RDW-CV**	%	11.0–16.0	41.0	11.0–17.0	27.6
**RDW-SD**	μm^3^	37.0–49.0	36.4	37.0–49.0	31.3
**PLT**	10^3^/μL	150–400	7.6	150–400	7.7
**WBC**	10^3^/μL	3.50–10.00	7.8	3.50–10.00	7.3
**LYM #**	10^3^/μL	1.00–3.00	12.2	1.00–3.00	12.6
**LYM %**	%	15.0–45.0	55.2	15.0–45.0	55.9
**MON #**	10^3^/μL	0.20–0.80	5.4	0.20–0.80	6.9
**MON %**	%	4.0–12.0	9.6	4.0–12.0	8.9
**NEU #**	10^3^/μL	1.60–7.00	29.6	1.60–7.00	24.4
**NEU %**	%	40.0–73.0	48.4	40.0–73.0	41.5
**EOS #**	10^3^/μL	0.00–0.50	19.2	0.00–0.50	15.2
**EOS %**	%	0.5–7.0	25.6	0.5–7.0	22.0
**BAS #**	10^3^/μL	0.00–0.15	4.2	0.00–0.15	3.9
**BAS %**	%	0.0–2.0	17.4	0.0–2.0	15.2

**Table 7:** Out of Range (OOR) biochemistry values based on comparison with DIALAB values.

Parameter	Unit	Male		Female	
		DIALAB values	%OOR	DIALAB values	%OOR
AST	U/L	0.0–37.0	0.2	0.0–31.0	3
ALT	U/L	0.0–41.0	0	0.0–31.0	3
ALP	U/L	53.0–128	0.2	42.0–98.0	3.3
GGT	U/L	0.0–55.0	1.8	0.0–38.0	9.3
BID	mg/dL	0.0–0.30	6.1	0.0–0.30	3.9
BIT	mg/dL	0.1–1.20	1.6	0.1–1.20	0.9
Albumin	g/dL	3.5–5.2	0.5	3.5–5.2	0.7
Prot-T	g/dL	6.6–8.3	11.1	6.6–8.3	10.2
TChol	mg/dL	0.0–200.0	11.3	0.0–200.0	10.4
Trig	mg/dL	0.0–160.0	0	0.0–160.0	0.5
HDL-C	mg/dL	35.30–79.50	0	42.0–88.0	5.3
LDL-C	mg/dL	0.0–130.0	6.3	0.0–130.0	3.3
Urea	mg/dL	8.0–23.0	5.2	8.0–23.0	3.3
Creatinine	mg/dL	0.90–1.30	36.8	0.60–1.10	15.1

## Discussion

Although evidence suggests that disparities in socio-demographic and genetic factors affect the development of RIs, the healthcare system in Ghana continues to depend on pre-established RIs that were developed from Caucasian populations. This study reports the haemato-biochemical RIs from four eco-geographical zones in Ghana, highlighting gender-based differences and disparities in comparison to the RIs that accompany the analyzers used, as well as RIs from other studies.

This study found the RIs for RBC, Hb, HCT, MCV and MCH to be higher in males compared to females whereas WBC and platelet counts were higher in females compared to males as consistent with previous reports in other African countries [8, 18–22]. The differences in RIs based on sex may be linked to the effect of menstruation and its associated increased demand for iron, differences in androgen hormones (testosterone and oestrogen) and the extent to which erythropoiesis and megakaryopoiesis are regulated in males and females [21, 23, 24].

Several variations between the RIs in this study and those provided by the manufacturer of the haematology analyzer were observed. The RIs for Hb level, HCT, MCV, MCH, MCHC were below the lower limit of the accompanying RIs. These parameters also presented with substantial misclassification, with %OOR ranging from 16.10%-35.8%, when compared to the accompanying RIs from the manufacturer. Other studies in Ghana [13] and other African countries [12, 25, 26] have highlighted similar disparities. This finding suggests that a proportion of normal participants whose haematological results are interpreted based on the pre-established (manufacturer’s) RIs may be erroneously classified as having anaemia.

The dropping of the lower limit of the red cell indices among the study population compared to the accompanying RIs may be attributed to the relatively lower ferritin and transferrin saturation among blacks [27] as well as poor nutritional status among the general Ghanaian population [28]. This highlights the influence of geographical variations on the normal physiology and support the need for the development of population-specific RI as mandated by the CLSI laboratory regulatory guidelines.

Furthermore, there were significant intra-population differences in the haematology RIs with respect to eco-geographic characteristics. The Transitional zone had the highest RBC, Hb and MCHC. This can be explained by the fact that the Transitional zone is mountainous (over 500 m above sea level) with conducive climate (optimal annual rainfall, temperature and humidity) and vegetation for agriculture. High altitude has been linked with increased erythropoiesis [29] and the predominant consumption of natural organic products (fruits and vegetables) compared to the other zones could also account for the relatively increased Hb. Furthermore, as consistent with our previous report [13], the Savannah zone had higher MON#, BAS# and BAS%. This finding could be linked to higher incidence of allergic conditions and parasitic infections in the Savannah zone [30].

This study also found significant gender-based and intra-population (based on eco-geography) variations in most of the biochemistry RIs. As coherent with previous studies in Ghana by Dosoo et al. [8] and Koram et al. [9] as well as studies by Abebe et al [31] in Ethiopia and Samaneka et al. [18] in Zimbabwe, the liver function analytes, kidney function analytes, and lipid profile (Trig and LDL-C) were higher among males compared to females. On the contrary, females had higher HDL-C compared to males. These findings were expected because males for instance have relatively greater skeletal muscle and bone mass which influences their serum creatinine. Additionally, the higher urea levels in males compared to females could be linked with the increased protein intake generally observed among males in the region. Of note, the Coastal Savannah had higher lipid profile whereas the Transitional zone had higher liver and kidney function analytes. These variations in biochemistry intervals could be attributed to differences in ethnic/genetic, nutritional and cultural/social lifestyle in the different eco-geographical zones [8, 9, 31, 32]. Importantly, the RIs of biochemistry analytes in this study were lower compared to the manufacturer’s values with %OOR ranging from 0.50%-15.10%. These variations in RIs may have undesirable results on clinical management of patients (i.e. misclassification and subsequent denial of appropriate care and treatment).

### Limitations

This study is limited by the fact that lipid samples were non-fasting. Of note, according to most national and international guidelines, RIs for lipid profiles are established on fasting blood samples when used for cardiovascular risk assessment. Additionally, because the study was limited to young adult blood donors, this study was unable to determine the RIs of children and older individuals. Due to the difficulty in standardizing dietary patterns based on international guidelines, the study was unable to directly assess them. Further studies are thus warranted. Furthermore, the study was conducted among apparently healthy blood donors and may not be reflective of the general population. Interpretation of the results should thus be approached with caution.

## Conclusion

This study established haematological and biochemistry RIs that would be potentially useful in the diagnosis, management and monitoring of disease progression in the study settings. There were variations in RIs within the population and between the study regions and other countries. Further local and nationwide studies are recommended to establish local and national RIs for haematological and biochemistry parameters.

## Supporting information

S1 File Data (haematology) and Data (biochemistry): Excel sheet of datasets on which the conclusions of this manuscript were made. (XLSX)

S1 Table Haematology reference intervals by gender. (XLSX)

S2 Table Haematology reference intervals by eco-geographical zones. (XLSX)

S3 Table Biochemistry reference intervals by gender. (XLSX)

S4 Table Biochemistry reference intervals by eco-geographical zones. (XLSX)
